# Pyloric stenosis as a manifestation of isolated gastric Crohn’s disease responding to intralesional steroid injection and balloon dilation: a case report

**DOI:** 10.1186/s13256-019-2272-8

**Published:** 2019-11-13

**Authors:** K. Balendran, S. Udumalagala, N. M. M. Nawaraththne

**Affiliations:** Gastroenterology & Hepatology unit, National Hospital of Sri Lanka, Colombo, 10 Sri Lanka

**Keywords:** Crohn’s disease, Isolated gastric Crohn’s disease, Pyloric stenosis

## Abstract

**Background:**

Crohn’s disease is a chronic inflammatory condition that can affect the gut from mouth to anus. Gastroduodenal involvement is seen in less than 5% of all patients with Crohn’s disease. Among those cases, isolated gastric Crohn’s disease is even rarer. Although most patients with isolated gastric involvement have nonspecific complaints, very few of them do develop features of pyloric obstruction. There is a paucity of data on specific management of gastric Crohn’s disease owing to its rarity and its frequent coexistence with colonic or ileal disease. We report a case of a patient who had pyloric stenosis as a manifestation of isolated gastric Crohn’s disease responding to intralesional steroid injection and balloon dilation.

**Case presentation:**

A previously healthy woman presented with recurrent postprandial vomiting, epigastric discomfort, and unintentional weight loss over 6 months. She had no diarrhea or extraintestinal manifestations. Clinically, she was pale and dehydrated. Examination of systems was unremarkable except for mild epigastric tenderness. Her initial laboratory findings were normocytic normochromic anemia, high inflammatory markers, and hypokalemia. Esophagogastroduodenoscopy revealed an inflamed pyloric mucosa with features of pyloric obstruction. Furthermore, magnetic resonance enterography confirmed the pyloric stenosis. Histopathological examination of a biopsy from the pylorus revealed noncaseating granuloma with superficial ulceration. Tuberculosis and sarcoidosis were excluded by appropriate investigations, and a diagnosis of gastric Crohn’s disease was made. Following the initial resuscitation, intralesional steroid injection and controlled radial expansion balloon dilation of the pylorus were carried out. The patient was commenced on azathioprine as a maintenance treatment, which led to a successful dilation and remarkable symptom improvement.

**Conclusion:**

Symptoms of pyloric obstruction as a manifestation of isolated gastric Crohn’s disease are extremely unusual in clinical practice, awareness of which would facilitate early appropriate investigations and treatment.

## Background

Crohn’s disease is a chronic inflammatory condition characterized by segmental involvement of the gastrointestinal tract from mouth to anus. The colon and ileum are the sites most commonly affected by Crohn’s disease [1]. Gastroduodenal involvement is seen in less than 5% of all patients with Crohn’s disease [2]. The vast majority of patients with gastroduodenal disease have simultaneous affectation of the terminal ileum or colon. Isolated gastric Crohn’s disease has been reported to occur in only 0.7% of all patients with Crohn’s disease [1]. The common presentation of isolated gastric Crohn’s disease includes epigastric discomfort, pain, nausea, weight loss, and constitutional symptoms. Pyloric stenosis is an extremely rare manifestation of isolated gastric Crohn’s disease. In adult patients with pyloric stenosis, other possible etiologies, such as malignancy, corrosive ingestion, and granulomatous diseases, should be ruled out [1, 3–8]. We report a unique case of a patient with pyloric stenosis as a manifestation of isolated gastric Crohn’s disease responding to intralesional steroid injection and balloon dilation.

## Case presentation

A 30-year-old Sri Lankan woman presented to the gastroenterology outpatient clinic of a tertiary care hospital with progressively worsening recurrent postprandial vomiting of 6 months’ duration. She also had epigastric discomfort, abdominal bloating, and unintentional weight loss of 7 kg. She denied abdominal pain, diarrhea, prolonged fever, rash, or joint pain. She had no medical or surgical comorbidities or history of acid/alkaline ingestion in the past. She had no family history of inflammatory bowel disease and no features of extraintestinal manifestations.

Clinical examination revealed an asthenic woman with mild dehydration and pallor. She had a temperature of 36.4 °C, respiratory rate of 16/minute, pulse rate of 90 beats/minute, and blood pressure of 100/60 mmHg. She had no orogenital ulcers, rashes, or evidence of peripheral or axial arthritis. Abdominal examination revealed epigastric tenderness without any palpable masses or organomegaly. Other system examinations were unremarkable.

Full blood count revealed normocytic normochromic anemia with hemoglobin of 9.2 g/dl, white cell count of 8500/mm^3^, and platelet count of 218,000/mm^3^. The patient had high inflammatory markers (erythrocyte sedimentation rate of 72 mm in the first hour, C-reactive protein of 58 mg/dl). She was hypokalemic with potassium of 3 mEq/L. Her renal, liver, and thyroid function and her cortisol and blood gases were within normal limits. She had normal calcium and angiotensin-convertingenzyme levels. Chest radiography was unremarkable for bilateral hilar lymphadenopathy or parenchymal abnormalities.

**Fig. 1:**
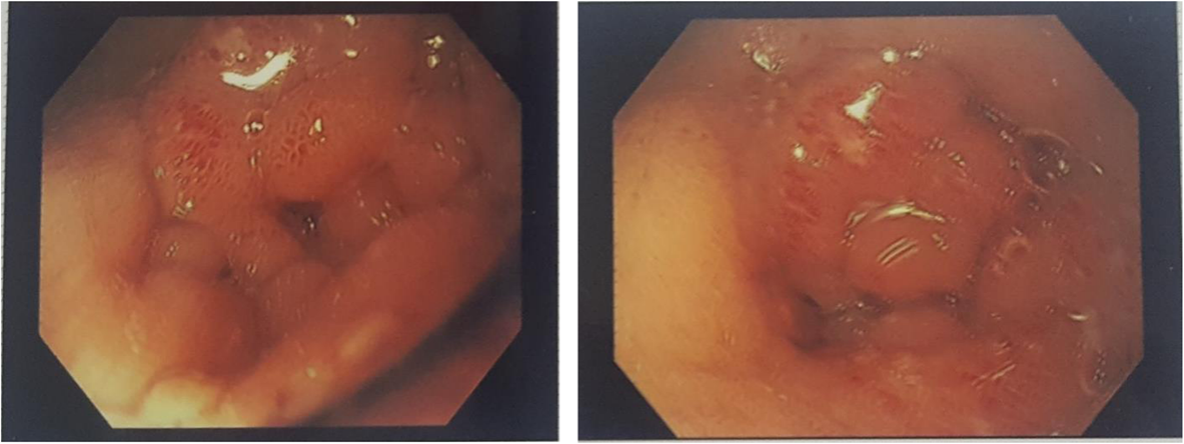
Esophagogastroduodenoscopy revealing inflamed edematous mucosa of pylorus

**Fig. 2:**
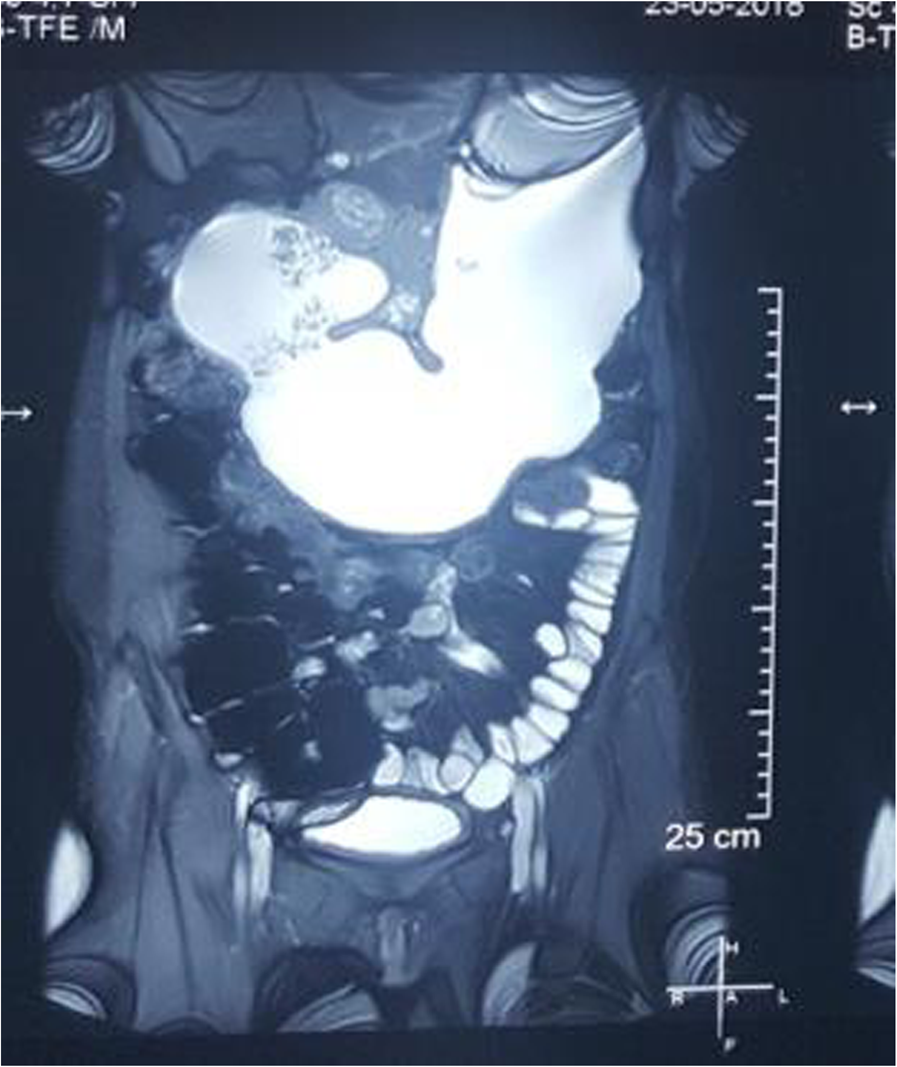
Magnetic resonance enterography revealing distended stomach with food particles and less mannitol in the small intestine

Esophagogastroduodenoscopy revealed inflamed edematous mucosa of the pylorus (Fig. 1) with features of pyloric obstruction, as evidenced by difficulty in negotiating the scope through a tight pylorus and food debris in the stomach despite 10 hours of fasting. A biopsy specimen was taken from the inflamed mucosa. Histopathological examination of the biopsy specimen showed moderate chronic inflammation with mucosal noncaseating granuloma and fissuring ulceration. No evidence of *Helicobacter pylori* was documented. The results of tuberculous culture and polymerase chain reaction from the biopsy specimen were negative. Magnetic resonance enteroclysis revealed a distended stomach with food particles indicating poor gastric emptying and decreased amount of mannitol in the small intestine (Fig. 2), suggestive of persistent gastric outlet obstruction. The result of her colonoscopy, which was done to rule out additional abnormalities in the distal ileum and colon, was normal.

The diagnosis of isolated gastric Crohn’s disease was made on the basis of clinical presentation, endoscopic findings, histopathological features, and exclusion of other causes of granulomatous gastritis, such as sarcoidosis and tuberculosis.

She was initially resuscitated with intravenous fluids and potassium. Intralesional steroid (triamcinolone) injection followed by dilation of the pylorus was carried out. A controlled radial expansion balloon dilator with inflation pressure of 9 atm (10 mmHg) was used to successfully dilate the narrowed pylorus. The patient was commenced on oral omeprazole and azathioprine 50 mg as a maintenance immunosuppressant. She showed a remarkable response at 4-week follow-up with complete resolution of her symptoms, and she started to gain weight as well.

## Discussion

This case report describes a case of a woman who presented with features of pyloric obstruction associated with isolated gastric Crohn’s disease successfully treated with intralesional steroid injection and balloon dilation. Management of isolated gastric Crohn’s disease can be divided into medical and surgical, which includes endoscopic, laparoscopic, and open surgical interventions. However, there is a paucity of data on specific management of gastric Crohn’s disease, owing to its rarity and frequent coexistence with colonic or ileal disease [1, 9].

In terms of medical management, various therapeutic agents have been tried. Systemic steroids remain a mainstay of treatment. One case report demonstrated the use of inhaled steroids in the management of isolated gastric Crohn’s disease [10]. Methotrexate, thiopurines, and 5-amino salicylic acids have been described to show some promising effects. However, there are no reports specific to isolated gastric Crohn’s disease. Oral proton pump inhibitors have been tried in a few cases. They provide symptomatic but not anti-inflammatory benefit in treating gastric Crohn’s disease [4]. The efficacy of infliximab (anti-tumor necrosis factor) therapy and calcineurin inhibitors for upper intestinal Crohn’s disease has been described in a single case report [11]. Another case report described successful use of adalimumab to treat a patient with gastric Crohn’s disease who presented with pyloric obstruction [2].

Endoscopic treatment options for pyloric stenosis include intralesional steroid injection, balloon dilation, and stent placement. There have been no controlled studies or evidence of effectiveness of these interventions in gastric Crohn’s disease. However, one case report revealed a remarkable response to balloon dilation in a patient who presented with pyloric stenosis associated with Crohn’s disease [1]. In addition to balloon dilation, we used an intralesional steroid, triamcinolone, which is an intermediate-acting synthetic glucocorticoid. Though it has been used to treat several inflammatory conditions, including ileocolonic Crohn’s disease [12], there are no data on its use in gastric Crohn’s disease. Because our patient showed a remarkable response to this treatment, we suggest combining intralesional steroid injection with balloon dilation as a safe way of treating pyloric stenosis associated with Crohn’s disease. Although our patient responded to endoscopic and medical therapy, she did not require any surgical interventions. Therefore, surgical therapy in gastric Crohn’s disease should be reserved for conditions such as massive bleeding, unsuccessful balloon dilation, or gastric fistula [1].

## Conclusion

Symptoms of pyloric obstruction as a manifestation of isolated gastric Crohn’s disease are extremely unusual in clinical practice, awareness of which would facilitate early appropriate investigations and treatment.

## Data Availability

Not applicable.
